# Occult Non-Small Cell Lung Cancer: An Underappreciated Disease

**DOI:** 10.3390/jcm11051399

**Published:** 2022-03-03

**Authors:** Jingsheng Cai, Fan Yang, Xun Wang

**Affiliations:** Department of Thoracic Surgery, Peking University People’s Hospital, Beijing 100044, China; caijs11@163.com

**Keywords:** non-small cell lung cancer, occult, prognosis, survival

## Abstract

Background: The number of researches on occult non-small cell lung cancer (NSCLC) is modest. Herein, we defined the clinicopathological features, prognosis and survival outcome of this underappreciated tumor, with purpose of obtaining a clearer picture on this disease. Methods: The entire cohort was categorized into two groups (occult NSCLC and other NSCLC) and further into five groups (occult, T1, T2, T3 and T4). A least absolute shrinkage and selection operator (LASSO) penalized Cox regression model was performed to identify the prognostic indicators. A nomogram and a risk-classifying system were formulated. Kaplan–Meier with Log-rank method was carried out to compare overall survival (OS) and cancer specific survival (CSS) differences between groups. Results: 59,046 eligible NSCLC cases (occult NSCLC: 1158 cases; other NSCLC: 57,888 cases) were included. Occult NSCLC accounted for 2.0% of the included cases. Multivariate analysis revealed that age, sex, tumor location, histology, grade and surgery were prognostic factors for OS. The corresponding prognostic nomogram classified occult NSCLC patients into low-risk and high-risk group, and its performance was acceptable. Survival curves demonstrated that occult NSCLC patients exhibited worse survivals than other NSCLC. In further analyses, the survival of low-risk occult NSCLC and stage T3 NSCLC were comparable, and the high-risk occult NSCLC patients still owned the worst survival rate. Conclusions: Occult NSCLC was an aggressive tumor with poor prognosis, and surgery was the preferred treatment. More attention should be paid to this overlooked disease due to no evidence of tumor imaging.

## 1. Background

Occult lung cancer, defined as the presence of malignant tumor cells in bronchial washing or sputum but demonstrated no tumor evidence by imaging [1,2], often manifests as metastasis diseases [3,4,5] or other internal diseases such as stroke [6,7,8,9], venous thromboembolism [10,11] and dermatomyositis [12]. When these patients develop apparent non-cancer-related symptoms, the vast majority may have already progressed to an advanced stage.

As per the current eighth tumor–node–metastasis (TNM) staging system, occult non-small cell lung cancer (NSCLC) is categorized as TxN0M0 [13]. Owing to the ambiguous TNM stage, the prognosis of this subgroup disease is still an enigma, and elucidation of prognostic indicators is therefore necessary to define high-risk patients who may derive benefit from more intensive care. Following extensive literature review, due to the low incidence (about 0.53%) [8] and lack of tumor evidence by imaging [1,2], the number of related researches is still modest, and most of them are case reports [3,4,5,7,8,9,11]. The lack of clinical data makes the natural course of occult NSCLC far from understood and the establishment of corresponding therapeutic strategies impossible, and it also compromises efforts to define prognostic factors for occult NSCLC.

Herein, given the paucity of data on prognosis and optimal management, the current study analyzed the data of occult NSCLC recorded in the Surveillance, Epidemiology, and End Results (SEER) database to more clearly define clinical features, prognosis and survival outcomes of this tumor, with purpose of providing further insights into this underappreciated disease.

## 2. Methods

### 2.1. Patient Selection

**Figure 1:**
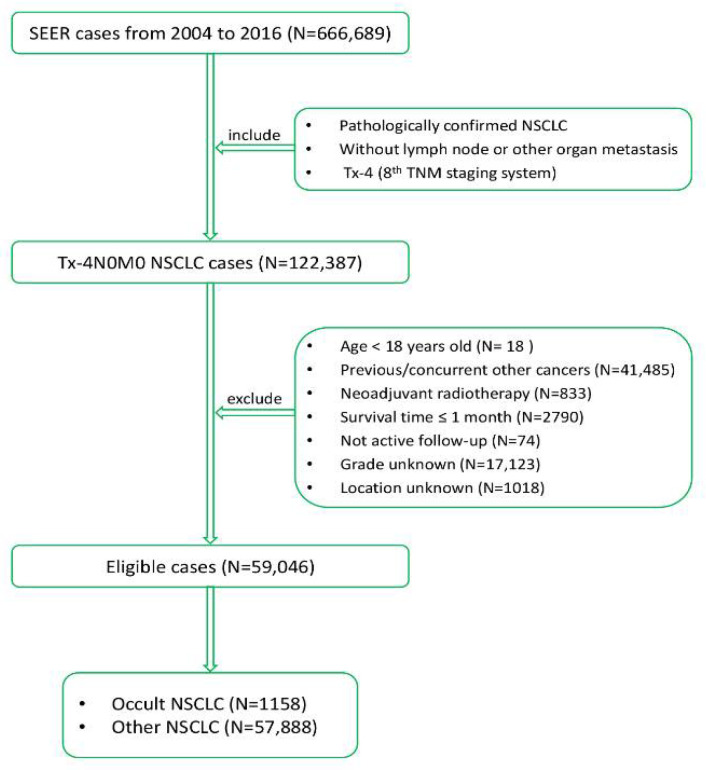
The study algorithm for patient enrollment in this study. SEER, Surveillance, Epidemiology, and End Results; NSCLC, non-small cell lung cancer; TNM, tumor–mode–metastasis.

From 2004 to 2016, patients diagnosed as NSCLC were reviewed from SEER database using SEER*Stat software version 8.3.4. The eligible cases fit the following criteria: (1) pathologically diagnosed as NSCLC; (2) without lymph node or other organ metastasis; (3) diagnosed as stage Tx-4 (the 8th TNM staging system). The exclusion criteria were: (1) age < 18 years old; (2) previous or concurrent other cancers; (3) received neoadjuvant radiotherapy; (4) survival time ≤ 1 month; (5) not active follow-up; (6) grade unknown; (7) location unknown. The entire cohort was divided into 2 subgroups: occult NSCLC and other NSCLC group, and further into five subgroups: Tx, T1, T2, T3 and T4 group. The study algorithm for patient enrollment is depicted in Figure 1.

Permission was obtained to retrieve SEER data files with the reference number: 12962-Nov2019. Due to the fact that patient data recorded in the SEER dataset have been de-identified, ethical approval and informed consent from individual patients were waived for this retrospective analysis.

### 2.2. Data Collection

The patients’ medical records were retrospectively reviewed for demographic information as well as for pertinent clinicopathological features including age (≤60 years old and >60 years old), sex (male and female), ethnicity (Caucasian, African and other), marital status (married and other), location (upper lobe, middle lobe, low lobe and other), surgery (no, lobectomy, pneumonectomy, sublobectomy, and other), radiotherapy (no and yes), chemotherapy (no and yes), histology (adenocarcinoma, squamous cell carcinoma, and other), grade (well differentiated, moderately differentiated, and poor differentiated/undifferentiated), status, and survival time. Complete data analyses were carried out in this study. According to the category of T stage. The eighth TNM staging system was applied in this study.

### 2.3. Follow-Up

Overall survival (OS) was defined as the time interval from the date of diagnosis to the date of death from any cause or the last known contact. Cancer specific survival (CSS) was calculated from the date of diagnosis to the date of death caused by the tumor or the date of the most recent follow-up. Patients included in this study had definitive survival status and exact survival time. The median follow-up time was 40 months (range from 2 to 155 months) in the entire cohort.

### 2.4. Statistical Analysis

Statistical analysis was performed by R version 4.1.1 (The R Foundation for Statistical Computing, Vienna, Austria; http://www.r-project.org (accessed on 10 September 2021)) and IBM SPSS Statistics (version 25.0, IBM Corp, Armonk, NY, USA). The Kaplan–Meier method was conducted to plot survival curves, and differences between survival curves were detected by log-rank test. The covariates including age, sex, ethnicity, marital status, tumor location, surgery, chemotherapy, radiotherapy, histology and grade were entered into a least absolute shrinkage and selection operator (LASSO) by using the R package “glmnet” and “lambda.1se” was used to select the variables. A forward stepwise multivariate Cox proportional hazard regression model was carried out to estimate the potential prognostic factor based on the results of LASSO. The nomogram, a tool which could integrate many prognostic indicators and predict the probability of an event more intuitive and convenient than traditional methods [14,15]. Based on the results of multivariate Cox analysis, a prognostic nomogram was established by using the R package “rms”. Harrell’s C-index [16] was performed to evaluate the performance of the nomogram. Using scaled line segments in the nomogram, various prognostic covariates are listed and scored, and the total score of all covariates can be used to predict the outcome [15]. X-tile software [17] was then used to dichotomize the total score into two subgroups (low-risk and high-risk). Categorical variables, provided as frequency and percentage, were compared by using Pearson χ2 test. Continuous variables, provided as mean and standard deviation (SD), and median and range, were compared by using Mann–Whitney U test. Two-sided of *p* < 0.05 was considered statistically significant.

## 3. Results

### 3.1. Patient Characteristics

From 2004 to 2016, 666,689 NSCLC cases from the SEER database were retrospectively investigated. The inclusion and exclusion criteria yielded a sample size of 59,046 cases (Tx/occult NSCLC group: 1158 cases; T1 group: 31,240 cases; T2 group: 16,223 cases; T3 group: 6749 cases; T4 group: 3676 cases). The study algorithm for patient enrollment is depicted in Figure 1.

The general demographic and clinicopathological characteristics of occult NSCLC and other NSCLC are summarized in Table 1. Regarding the occult NSCLC, the incidence of this subgroup tumor was about 2.0% (1158/59,046). The median age was 73 years old (rang from 33 to 95 years old). Over half of the include cases were male (54.1%). Caucasian constituted the majority of the entire cohort (80.7%). Most tumors located in the UL (55.4%). Only 15.7% of patients received surgery, 25.6% received chemotherapy, and 2.4% received radiotherapy. More patients were diagnosed with poor/undifferentiated tumors (42.1%). In contrast to occult NSCLC, more patients in other NSCLC group were younger (*p* < 0.001) and performed surgery (*p* < 0.001).

### 3.2. LASSO Penalized Multivariate Cox Regression Analysis

**Figure 2:**
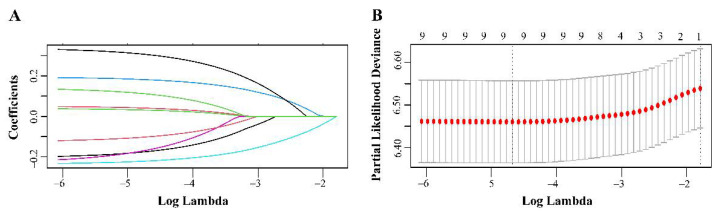
Prognostic indicators selection using the LASSO model analysis. LASSO coefficient profiles of nine covariates against the log (Lambda) sequence for OS (**A**). tuning parameter (Lambda) selection in the LASSO model used 10-fold cross-validation via minimum criteria for OS (**B**). LASSO, least absolute shrinkage and selection operator; OS, overall survival.

A LASSO regression model analysis was used to screen out the prognostic factors of occult NSCLC with the best predictive performance using the 10-fold cross-validation (Figure 2). Nine variables, including age, sex, ethnicity, marital status, tumor location, surgery, histology, grade, and radiotherapy, were entered into the multivariate Cox analysis based on the results of LASSO regression model. The multivariate Cox analysis confirmed that age (HR = 1.277, 95% CI 1.057–1.544, *p* = 0.011), sex (HR = 0.849, 95% CI 0.739–0.975, *p* = 0.020), location (HR = 1.219, 95% CI 1.062–1.399, *p* = 0.013), surgery (HR = 0.310, 95% CI 0.235–0.408, *p* < 0.001), histology (HR = 1.220, 95% CI 1.167–1.691, *p* = 0.006), and grade (HR = 1.376, 95% CI 1.129–1.677, *p* = 0.002) were independent prognostic factors for OS (Table 2).

### 3.3. Nomogram and Risk-Classifying System

**Figure 3:**
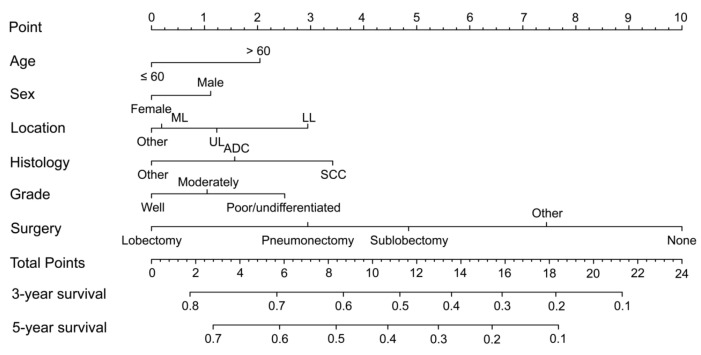
Nomogram for predicting the 3-year and 5-year overall survival in occult NSCLC patients. UL, upper lobe; ML, middle lobe; LL, low lobe; ADC, adenocarcinoma; SCC, squamous cell carcinoma.

A prognostic nomogram, based on the results of multivariate Cox regression analysis, was formulated (Figure 3). The nomogram revealed that surgery weighted more in determination of prognosis when compared with some other inherent tumor characteristics such as histology and grade. The C-index of the nomogram was 0.65 (95% CI: 0.63–0.67). Using scaled line segments in the nomogram, each covariate had a corresponding score (Table 3). A cutoff value (15) was obtained through the X-tile software, and the total score was dichotomized into two categories (low-risk occult NSCLC and high-risk occult NSCLC group). There were 278 cases and 880 cases in the low-risk and high-risk groups, respectively.

### 3.4. Survival Analysis

**Figure 4:**
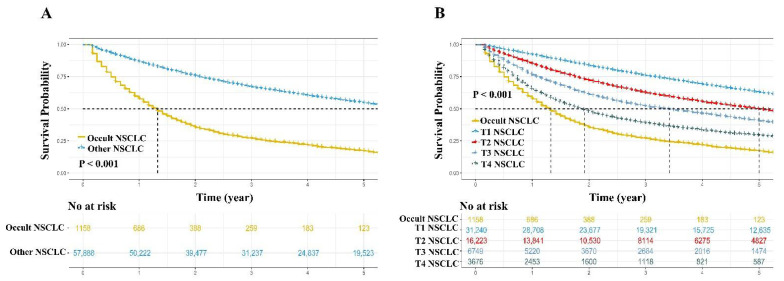
The overall survival comparisons. Occult NSCLC vs. other NSCLC (**A**) and occult NSCLC vs. T1 stage NSCLC vs. T2 stage NSCLC vs. T3 stage NSCLC vs. T4 stage NSCLC (**B**). NSCLC, non-small cell lung cancer.

When comparing with other NSCLC, the occult NSCLC showed worse survival rate (5-year OS rate: 17.2% vs. 54.5%, *p* < 0.001, Figure 4A; 5-year CSS rate: 25.7% vs. 66.5%, *p* < 0.001, Figure S1A). In further analyses, other NSCLC was categorized into four subgroups (T1, T2, T3, and T4 groups) based on the T stage, and the survival curves demonstrated that occult NSCLC still exhibited the worst survival time, followed by a progressive degradation of OS depending on T stage (OS: *p* < 0.001, Figure 4B; CSS: *p* < 0.001, Figure S1B).

**Figure 5:**
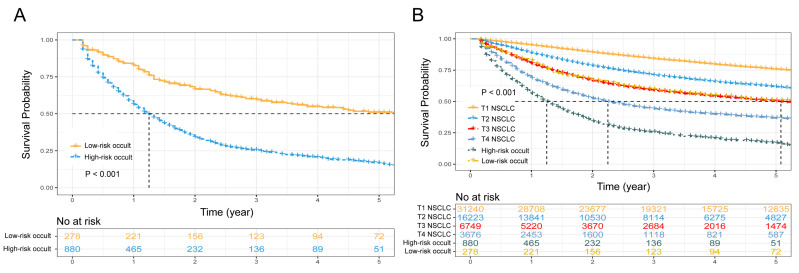
The overall survival comparisons. Low-risk occult NSCLC vs. High-risk occult NSCLC (**A**) and Low-risk occult NSCLC vs. High-risk occult NSCLC vs. T1 stage NSCLC vs. T2 stage NSCLC vs. T3 stage NSCLC vs. T4 stage NSCLC (**B**). NSCLC, non-small cell lung cancer.

The risk-classifying system assigned occult NSCLC patients into the low-risk group and the high-risk group. Patients in the low-risk group had extended survival time than patients in the high-risk group (5-year OS rate: 40.4% vs. 9.8%, *p* < 0.001, Figure 5A; 5-year CSS rate: 51.0% vs. 16.7%, *p* < 0.001, Figure S2A). Furthermore, the low-risk occult NSCLC patients and T3 stage patients had almost overlapping survival curves (5-year OS rate: 40.4% vs. 40.6%, *p* < 0.001, Figure 5B; 5-year CSS rate: 51.2% vs. 51.0%, *p* < 0.001, Figure S2B), and the high-risk occult patient had the least survival rate (OS: Figure 5B; CSS: Figure S2B).

**Figure 6:**
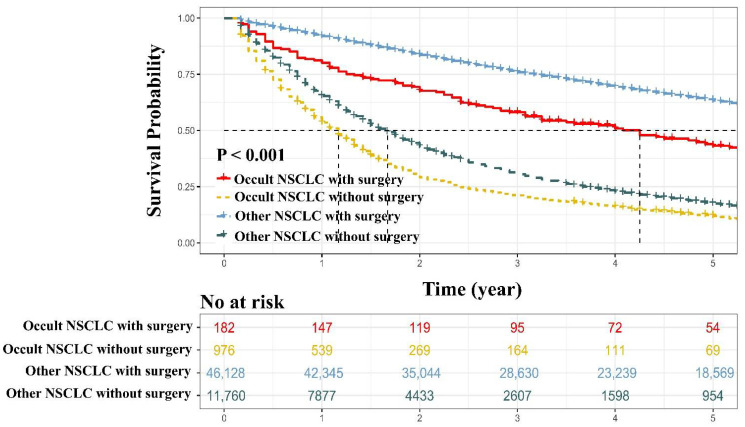
The overall survival comparisons. Occult NSCLC with surgery vs. occult NSCLC without surgery vs. other NSCLC with surgery vs. other NSCLC without surgery. NSCLC, non-small cell lung cancer.

On the subset analysis, the survival curves displayed that surgical resection brought great survival benefit to occult NSCLC cases (5-year OS rate: occult NSCLC with surgery vs. occult NSCLC without surgery = 43.2% vs. 12.1%, *p* < 0.001, Figure 6; 5-year CSS rate: occult NSCLC with surgery vs. occult NSCLC without surgery = 55.8% vs. 19.0%, *p* < 0.001, Figure S3). In addition, regarding the resected cases, occult NSCLC still showed a significantly increased mortality risk compared with other NSCLC (5-year OS rate: occult NSCLC with surgery vs. other NSCLC with surgery = 43.2% vs. 63.4%, *p* < 0.001, Figure 6; 5-year CSS rate: occult NSCLC with surgery vs. other NSCLC with surgery = 55.8% vs. 76.1%, *p* < 0.001, Figure S3). Accordingly, occult NSCLC had lower survival rate compared with other NSCLC among patients without surgery (5-year OS rate: occult NSCLC without surgery vs. other NSCLC without surgery = 12.1% vs. 18.0%, *p* < 0.001, Figure 6; 5-year OS rate: occult NSCLC without surgery vs. other NSCLC without surgery = 19.0% vs. 28.1%, *p* < 0.001, Figure S3).

## 4. Discussion

In this study, a large series of occult NSCLC was investigated. The findings of this research can be summarized as follows. First, occult NSCLC accounted for 2.0% of the included cases, and most of them did not receive surgery. Second, LASSO penalized multivariate Cox analyses revealed that age, sex, tumor location, grade, histology, and surgery were independent statistically significant prognostic factors. Third, a prognostic nomogram, developed based on the results of multivariate Cox analysis, classified occult NSCLC patients into low-risk and high-risk group, and its performance was acceptable. At last, occult NSCLC patients exhibited worse survival rates than other NSCLC. The survival of low-risk occult NSCLC patients and stage T3 NSCLC were comparable, and the high-risk occult NSCLC patients had the worst survival rate. Furthermore, surgical resection conferred survival benefit to the occult NSCLC patients. Our study might inform that more attention should be paid to the occult NSCLC patients who was overlooked due to no tumor evidence of imaging.

It is known that the eighth TNM staging system, the dominating prognostic factor for patient survival, was used to guide clinical decision making [2,13]. Therefore, an exact TNM stage could undoubtedly assist in administering proper treatment strategy to NSCLC patients and extend patients’ survival. Regarding occult NSCLC however, this population was categorized as TxN0M0 [13]. Therefore, it was deemed to be plausible to evaluate other prognostic factors apart from the variables of TNM stage to predict the prognosis of this population.

In our study, LASSO model analysis was applied to select the prognostic factors, which helped to avoid overfitting of the model. After that, prognostic factors, selected from the LASSO model, were entered into the multivariate Cox analysis. Finally, a nomogram was established. As is known that nomogram is a widely used and efficient tool for predicting prognosis in malignancies such as breast cancer [18], gastric cancer [19], esophageal carcinoma [20], and lung cancer [21,22,23]. The C-index of our nomogram was little pale (only 0.65). A possible explanation for this was that the major prognostic factors such as tumor size, lymph node status and distant metastasis status could not be determined in the occult NSCLC and thus were not included in this nomogram. With the help of this nomogram, the occult NSCLC patients were divided into two categories (low-risk and high-risk), and the low-risk occult NSCLC had superior survival rate than the high-risk occult NSCLC. From our perspectives, more emphasize should be put on the medical care of these patients, especially the high-risk ones. With the help of the nomogram, clinicians might could estimate personal survival and guide treatment strategy selection. To be more specific, the high-risk occult NSCLC patients may should select the more intensive scheduled follow up strategy or even additional therapies. The low-risk occult NSCLC patients could be treated as the local tumors (T3N0M0, stage IIB).

In the study by Wu et al. [24], the authors investigated 2353 occult lung cancer cases from the SEER database and demonstrated that the CSS of the occult lung cancer was comparable to that of the stage T3 lung cancer. In their study however, small cell lung cancer was also included. It is known that the natural course, prognosis and survival of small cell lung cancer were dramatically different from NSCLC. Therefore, it was advisable to study these two kinds of lung cancers separately. In our study, only NSCLC was enrolled, and the survival curves indicated that the occult NSCLC conferred the worst OS rate when comparing with other NSCLC. It may be speculated that although the imaging does not prove evidence of tumor, these patients may already have reginal lymph nodes or distant organs micrometastasis. In addition, previous researches implied that occult NSCLC often manifests as venous thromboembolism [10,11], which demonstrated that hematogenous metastasis occurred in this kind of NSCLC tumors. Undoubtedly, occult NSCLC had poor prognosis. Moreover, when compared with Wu et al.’s study, complete data analysis was performed in our study to minimize bias. More rigor and efficient statistical methodology-for example LASSO penalized multivariate Cox regression model and visualized nomogram made our results more reliable and readable.

Our results demonstrated that surgery type was the most predominating prognostic factor for occult NSCLC, and lobectomy have led to impressive gains in survival for these patients. In contrast, patients who were not performed surgery had the worst survival. The results were also confirmed in Wu et al.’s study [24], Bechtel et al.’s study [25] and Cortese et al.’s study [26]. Our finding was important for clinical practice. Due to no tumor evidence under imaging screening, clinicians might recommend systemic chemotherapy for these patients. As we all know that surgical resection is the mainstay treatment of early stage and local advanced NSCLC. Bronchial washing or sputum cytology positivity does not mean that the tumor is unresectable. Thus, missing the suitable surgical timing might have an adverse impact on patient’s survival.

Considering the poor prognosis of occult NSCLC, it is advisable to investigate the strategies for early detection of occult NSCLC and associated clinical features. It is known that occult NSCLC patients often manifest as the presence of malignant tumor cells in bronchial washing or sputum but with no tumor evidence by ordinary imaging [1,2]. Therefore, more efficient screen method for example positron emission tomography-computed tomography (PET-CT) might contribute to early detection of primary tumor. Clinicians need to be vigilant when they encounter patients who manifest as internal diseases such as stroke [6,7,8,9], thromboembolism [10,11] and dermatomyositis [12]. As occult NSCLC might be the fundamental cause of these non-cancer-related symptoms, the more refined examinations should be performed as soon as possible.

This study had some limitations. Owing to the limited information recorded in the SEER database, some other novel efficient prognostic factors such as smoking history, pulmonary function, pathologic subtypes, spread through air space features, driver gene mutations, and tumor mutation burden were not included in this study. Further effort on broader geographic and clinicopathological characteristics recruitment are encouraged to improve this study. Besides, our results should be cautiously interpreted due to the retrospective nature of this study. The low incidence of occult NSCLC made it hard to validate our results in an independent cohort, and future other prospective studies are warranted to validate our conclusions.

## 5. Conclusions

In conclusion, our comprehensive analysis of occult NSCLC demonstrated that occult NSCLC was an aggressive tumor with poor prognosis, and surgery was the preferred treatment.

## Figures and Tables

**Table 1:** The clinicopathological features of included case.

Characteristics	Occult NSCLC (*N* = 1158)	Other NSCLC (*N* = 57,888)	*p*
	No. of Patients (%)	No. of Patients (%)	
Age, year			
Mean ± SD	71.9 ± 10.5	68.7 ± 10.1	<0.001 ^a^
Median (range)	73 (33–95)	69 (18–98)	
≤60	181 (15.6)	11,852 (20.5)	<0.001
>60	977 (84.8)	46,036 (79.5)	
Sex			<0.001
Male	627 (54.1)	28,108 (48.6)	
Female	531 (45.9)	29,780 (51.4)	
Ethnicity			<0.001
Caucasian	934 (80.7)	48,700 (84.1)	
African	151 (13.0)	5271 (9.1)	
Other ^b^	73 (6.3)	3917 (6.8)	
Marital status			<0.001
Married	553 (47.8)	31,431 (54.3)	
Other	605 (52.2)	26,457 (45.7)	
Location			<0.001
UL	642 (55.4)	35,138 (60.7)	
ML	65 (5.6)	2985 (5.2)	
LL	401 (34.6)	18,520 (32.0)	
Other	50 (4.3)	1245 (2.2)	
Surgery type			<0.001
None	976 (84.3)	11,760 (20.3)	
Lobectomy	109 (9.4)	36,250 (62.6)	
Pneumonectomy	2 (0.2)	1073 (1.9)	
Sublobectomy	32 (2.8)	8206 (14.2)	
Other	39 (3.4)	599 (1.0)	
Histology			<0.001
ADC	548 (47.3)	33,583 (58.5)	
SCC	551 (47.6)	19,414 (33.5)	
Other	59 (5.1)	4621 (8.0)	
Grade			0.001
Well	225 (19.4)	10,481 (18.1)	
Moderate	446 (38.5)	25,565 (44.2)	
Poor/undifferentiated	487 (42.1)	21,842 (37.7)	
Radiotherapy			<0.001
No	1130 (97.6)	55,051 (95.1)	
Yes	28 (2.4)	2837 (4.9)	
Chemotherapy			<0.001
No	862 (74.4)	47,861 (82.7)	
Yes	296 (25.6)	10,027 (17.3)	

^a^ Mann–Whitney U test; ^b^ other includes American Indian, Alaska native, Asian and pacific islander SD, standard deviation; UL, upper lobe; ML, middle lobe; LL, low lobe; ADC, adenocarcinoma; SCC, squamous cell carcinoma; NSCLC, non-small cell lung cancer.

**Table 2:** LASSO-penalized multivariate Cox regression analysis of OS.

Characteristics	Multivariate Analysis		
	HR	95% CI	*p*
Age, year			0.011
≤60	1		
>60	1.277	1.057–1.544	
Sex			0.020
Male	1		
Female	0.849	0.739–0.975	
Ethnicity			0.112
Caucasian	1		
African	0.876	0.723–1.061	
Other ^b^	0.784	0.592–1.038	
Marital status			0.215
Married	1		
Other	1.091	0.951–1.253	
Location			0.013
UL	1		
ML	0.893	0.655–1.217	
LL	1.219	1.062–1.399	
Other	0.880	0.633–1.224	
Surgery type			<0.001
None	1		
Lobectomy	0.310	0.235–0.408	
Pneumonectomy	0.440	0.109–1.774	
Sublobectomy	0.545	0.355–0.836	
Other	0.737	0.512–1.061	
Histology			0.006
ADC	1		
SCC	1.220		
Other	0.822	0.586	
Grade			0.002
Well	1		
Moderate	1.147	0.940–1.396	
Poor/undifferentiated	1.376	1.129–1.677	
Radiotherapy			0.506
No	1		
Yes	0.861	0.555–1.337	
Chemotherapy ^a^			
No			
Yes			

^a^ Chemotherapy was not included in the multivariate Cox analysis due to the LASSO model selection. ^b^ other includes American Indian, Alaska native, Asian, and Pacific Islander. LASSO, least absolute shrinkage and selection operator; OS, overall survival; UL, upper lobe; ML, middle lobe; LL, low lobe; ADC, adenocarcinoma; SCC, squamous cell carcinoma.

**Table 3:** Prognostic score of each covariate calculated based on the nomogram.

Characteristics	Category	Score
Age	≤60	0
	>60	2
Sex	Male	1
	Female	0
Location	UL	1
	ML	0
	LL	3
	Other	0
Surgery	None	10
	Lobectomy	0
	Pneumonectomy	3
	Sublobectomy	5
	Other	7
Grade	Well	0
	Moderate	1
	Poor/undifferentiated	3
Histology	ADC	2
	SCC	3
	Other	0
Risk-classifying system (cutoff value = 15)		
Low-risk	≤15	
High-risk	>15	

UL, upper lobe; ML, middle lobe; LL, low lobe; ADC, adenocarcinoma; SCC, squamous cell carcinoma.

## Data Availability

The dataset generated for this study is available in the SEER database (https://seer.cancer.gov/ (accessed on 10 September 2021)) which is available for the researchers who are authenticated.

## Supplementary Materials

The following supporting information can be downloaded at: https://www.mdpi.com/article/10.3390/jcm11051399/s1, Figure S1. The cancer specific survival comparisons. Occult NSCLC vs. other NSCLC (A) and occult NSCLC vs. T1 stage NSCLC vs. T2 stage NSCLC vs. T3 stage NSCLC vs. T4 stage NSCLC (B). NSCLC, non-small cell lung cancer. Figure S2. The cancer specific survival comparisons. Low-risk occult NSCLC vs. High-risk occult NSCLC (A) and Low-risk occult NSCLC vs. High-risk occult NSCLC vs. T1 stage NSCLC vs. T2 stage NSCLC vs. T3 stage NSCLC vs. T4 stage NSCLC (B). NSCLC, non-small cell lung cancer. Figure S3. The cancer specific survival comparisons. Occult NSCLC with surgery vs. occult NSCLC without surgery vs. other NSCLC with surgery vs. other NSCLC without surgery. NSCLC, non-small cell lung cancer.

## Abbreviations

NSCLC	non-small cell lung cancer
LASSO	least absolute shrinkage and selection operator
OS	overall survival
TNM	tumor-node-metastasis
SEER	Surveillance
HR	hazard ratio
CI	confidential interval
SD	standard deviation
UL	upper lobe
ML	middle lobe
LL	low lobe
ADC	adenocarcinoma
SCC	squamous cell carcinoma
