# Supplementary material for: Occult Non-Small Cell Lung Cancer: An Underappreciated Disease
Source: J Clin Med. 2022 Mar 3;11(5):1399. doi: 10.3390/jcm11051399 (PMC8910858; doi:10.3390/jcm11051399)
Supplement: Supplementary file 1 [file jcm-11-01399-s001.zip › figure legend of supplement figuures.pdf]

FIGURE LEGEND

Figure S1. The cancer specific survival comparisons. Occult NSCLC vs. other NSCLC (**A**) and occult NSCLC vs. T1 stage NSCLC vs. T2 stage NSCLC vs. T3 stage NSCLC vs. T4 stage NSCLC (**B**). NSCLC, non-small cell lung cancer

Figure S2. The cancer specific survival comparisons. Low-risk occult NSCLC vs. High-risk occult NSCLC (**A**) and Low-risk occult NSCLC vs. High-risk occult NSCLC vs. T1 stage NSCLC vs. T2 stage NSCLC vs. T3 stage NSCLC vs. T4 stage NSCLC (**B**). NSCLC, non-small cell lung cancer

Figure S3. The cancer specific survival comparisons. Occult NSCLC with surgery vs. occult NSCLC without surgery vs. other NSCLC with surgery vs. other NSCLC without surgery. NSCLC, non-small cell lung cancer
