# Supplementary figures and images for: Occult Non-Small Cell Lung Cancer: An Underappreciated Disease
Source: J Clin Med. 2022 Mar 3;11(5):1399. doi: 10.3390/jcm11051399 (PMC8910858; doi:10.3390/jcm11051399)

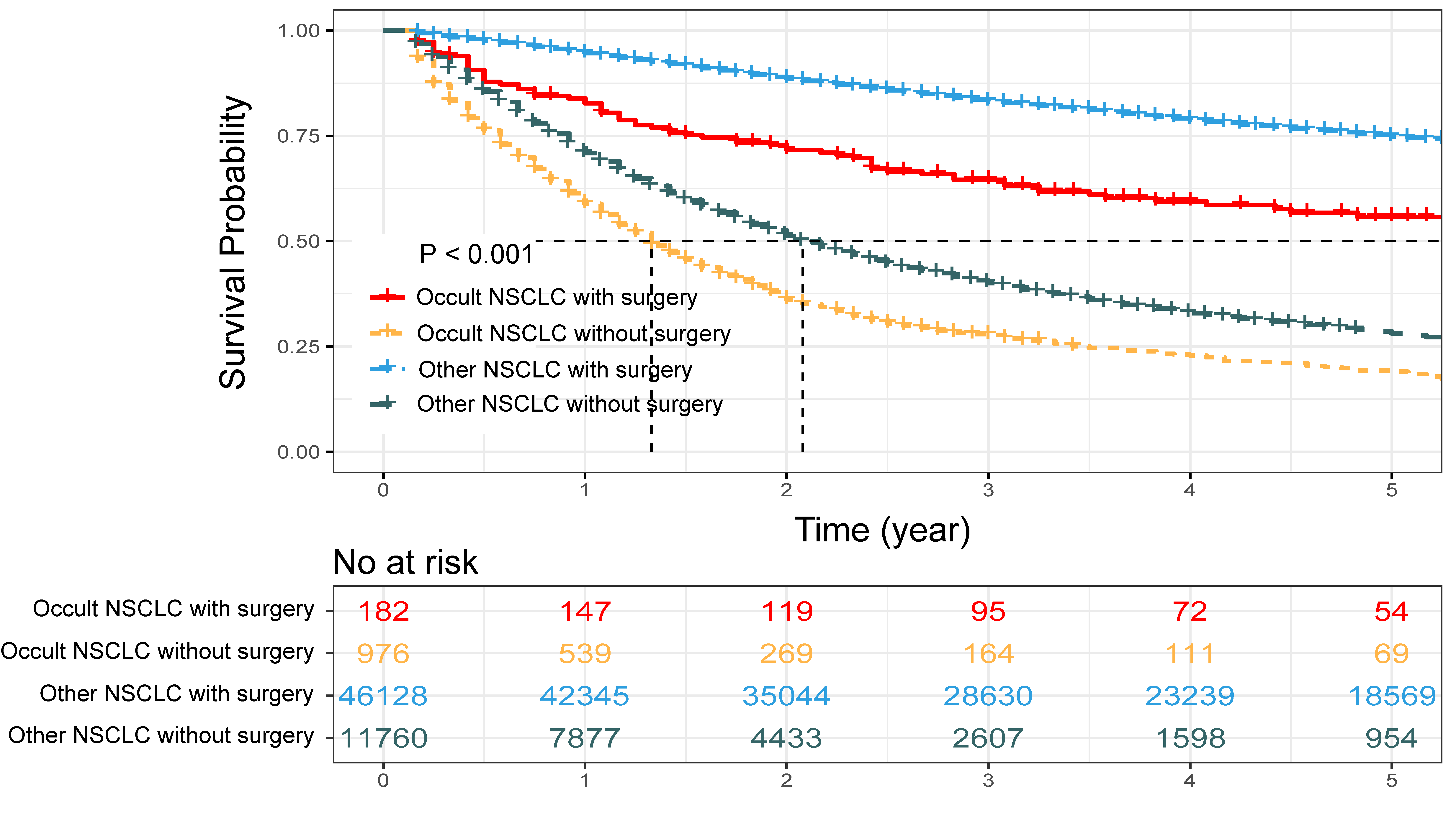

Supplement: Supplementary file 1 [file jcm-11-01399-s001.zip › Figure S3.tif]
